# The effect of acute taurine ingestion on 4-km time trial performance in trained cyclists

**DOI:** 10.1007/s00726-016-2282-4

**Published:** 2016-07-05

**Authors:** Ryan Ward, Craig A. Bridge, Lars R. McNaughton, S. Andy Sparks

**Affiliations:** Department of Sport and Physical Activity, Edge Hill University, Ormskirk, Lancashire UK

**Keywords:** Cycling, Buffering, Ergogenic aid, Performance

## Abstract

Taurine (TAU) has been shown to improve exercise time to exhaustion and 3-km running performance; however, no studies have considered the effect of acute TAU ingestion on short duration cycling time trial (TT) performance. The aim of this study was to determine the effects of a single oral acute dose of 1000 mg of TAU on a laboratory simulated 4-km cycling TT. Eleven trained male cyclists performed three, 4-km TTs. The first of the trials was a familiarisation, followed by two subsequent trials which were performed two hours after the consumption of either 1000 mg of TAU or placebo (P), using a double-blind randomised crossover design. Capillary blood samples were obtained prior to the start and immediately after each TT for the measurement of lactate, pH and HCO_3_^−^. There was no effect of TAU (*p* = 0.731, *d* = 0.151) on performance (390 ± 27 and 388 ± 21 s for TAU and P, respectively), nor were there any condition main effects for VO_2_, lactate, pH, or HCO_3_^−^ (*p* > 0.05) despite post TT changes in lactate (7.3 ± 2.5 mmol l^−1^, *p* < 0.001, *d* = 2.86, 7.6 ± 2.0 mmol l^−1^ *p* < 0.001, *d* = 3.75); pH (−0.255 ± 0.1, *p* < 0.001, *d* = 2.62, −0.258 ± 0.09, *p* < 0.001, *d* = 2.87); HCO_3_^−^ (−13.58 ± 2.7 mmol l^−1^, *p* < 0.001, *d* = 5.04 vs. −13.36 ± 2.3, *p* < 0.001, *d* = 5.72 for TAU and P, respectively). The findings of this study suggest that a pre-exercise dose of 1000 mg TAU offers no performance advantage during 4-km TT nor does it alter the blood buffering responses in trained cyclists.

## Introduction

Taurine (TAU) plays a role in a wide variety of physiological functions and is found in many tissues (Schaffer et al. 2010). Relatively few studies have however, explored its role in human physiological interventions. Taurine is abundant in skeletal muscle (Pierno et al. 2012), but its specific function is not currently fully understood. It has been suggested that TAU plays a role in aerobic exercise (Ward et al. 1999) and mitochondrial function (Hansen et al. 2010) since higher concentrations have been reported in the skeletal muscle of trained (~64 mmol kg^−1^ dw) vs. untrained (~50 mmol kg^−1^ dw) participants (Graham et al. 1995). In addition, Harris et al. (1998) have reported the TAU content of Type I muscle fibres of the vastus lateralis to be 39.2 ± 17.8 mmol kg^−1^ dw compared to 9.6 ± 2.6 mmol kg^−1^ dw in the Type II fibres of humans. In rats, higher concentrations of TAU have also been reported in the soleus compared to the gastrocnemius muscles (Matsuzaki et al. 2002), likely due to a higher proportion of Type I fibres usually observed in the soleus. In spite of there being some suggestions relating to the function of TAU, it remains unclear how different skeletal muscle fibre types might be affected either intra- or extracellularly by supplementation. Furthermore, it is also unclear how TAU supplementation might affect exercise performance in activities which recruit different proportions of either type I or type II fibres. The lack of observed increases in skeletal muscle TAU content with supplementation, suggest that it is the alterations to extracellular TAU concentration that may explain the previously reported performance improvements following acute ingestion (Balshaw et al. 2013).

In the plasma, TAU concentrations have been shown to increase following acute exercise (Cuisinier et al. 2001, 2002) with a suggestion that the rate of increase is directly related to exercise intensity (Ward et al. 1999). Cuisinier et al. (2001) suggested that increases in plasma TAU may be the result of skeletal muscle damage since it increased concomitantly with plasma creatine kinase concentrations. Studies that have reported increases in plasma TAU during exercise have used trained or well-trained participants with VO_2peak_ values of >50 ml kg min^−1^ (Cuisinier et al. 2001, 2002; Ward et al. 1999). In contrast, Galloway et al. (2008) reported that 4.98 g day^−1^ of TAU for 7 days had no effect on plasma or skeletal muscle TAU concentrations in active participants (VO_2peak_ ~40 ml kg min^−1^). Those studies that have measured muscle TAU concentrations have shown equivocal findings, likely the result of heterogeneous participant populations. It has been suggested that improvements in performance might be explained by either, an interaction between TAU and the muscle membrane, enhanced mitochondrial buffering (Hansen et al. 2006), or improved force production. These potential mechanisms may be mediated through increased calcium release from the sarcoplasmic reticulum leading to improved contractile filament sensitivity (Dutka et al. 2014).

The majority of studies that have investigated the role of exogenous TAU ingestion have used animal models with typically much higher doses those reported in human studies (Goodman et al. 2009). In humans, many of the previous studies that have administered exogenous TAU prior to exercise, have done so as part of a commercially available energy drink (Red Bull™) which also contains other active ingredients (Forbes et al. 2007; Alford et al. 2001; Geiss et al. 1994). This makes it difficult to ascertain specific responses to singularly ingested TAU. Of the human studies that have focused solely on TAU ingestion prior to exercise (Balshaw et al. 2013; Rutherford et al. 2010; Galloway et al. 2008; Zhang et al. 2004), only Galloway et al. (2008) reported no effects on either exercise performance (120 min at 60 % VO_2peak_) or on the metabolic responses following prolonged TAU supplementation. Rutherford et al. (2010) observed increased fat oxidation rates following acute ingestion of 1.66 g of TAU, but this was during a 90 min exercise bout at 65 % VO_2max_, that preceded a 5 kJ kg BM^−1^ time trial (TT), during which no changes in performance were observed. The authors postulate that TAU mediated increases in lipolysis, were the result of increased cyclic adenosine monophosphate, caused by elevated adenylyl cyclase activation (Rutherford et al. 2010; Spriet and Whitfield 2015). Conversely, Zhang et al. (2004) showed improved VO_2max_, exercise capacity, and maximal workloads following 7 days of 6 g day^−1^ of TAU. The only study to use a TT as a sole performance criterion was that of Balshaw et al. (2013) who demonstrated a 1.3 % improvement in 3-km TT running performance in well-trained middle distance athletes, following the acute ingestion of 1000 mg TAU.

Given the confounding nature of these data, it remains unclear how oral TAU supplementation may alter skeletal muscle function and improve performance. Taurine has previously been shown to improve lactic acid buffering (Nakada et al. 1991) in brain cells and intramuscularly in rats. However, in human exercise performance studies, post exercise plasma lactate has remained largely unaltered by TAU supplementation (Lee et al. 2003; Rutherford et al. 2010), even in the presence of an observed improvement in performance (Balshaw et al. 2013). Observations such as these, suggest that exogenously mediated increases in plasma TAU concentration, may influence skeletal muscle function, either by altering buffering or via changes to taurine flux. If these suggested mechanisms of TAU action are correct, short duration, high intensity exercise is of particular interest, due to its profound effects on acid–base balance (Fitts 2008). Taurine is frequently included as an active ingredient in both sport and stimulant drinks, but at present, no studies have considered the effects of these types of acute doses of TAU on short duration cycling TT performance. Although there is some evidence from rat studies to suggest that there may be improved calcium regulation in skeletal muscle (Bakker and Berg 2002), it has yet to be demonstrated if TAU acts to alter extracellular buffering during high intensity exercise in trained human participants. Therefore, the aims of the present study were (1) to determine the effects of an ecologically relevant acute oral dose of TAU, on a laboratory simulated 4-km cycling TT performance in trained cyclists and (2) to determine the effects this ingestion strategy has on blood acid–base responses.

## Method

Following ethical approval, health screening and informed consent, 11 male trained (De Pauw et al. 2013) cyclists (mean ± SD age 34.6 ± 11.5 years, height 178.1 ± 5.2 cm, weight 74.8 ± 6.2 kg and body fat 15.5 ± 4.6 %, training >3 h week^−1^ and at least 2 years’ racing experience) performed three, 4-km TTs on a CompuTrainer Pro cycle ergometer (RacerMate, Seattle, USA). The CompuTrainer was fitted with each participants own bicycle, which was then calibrated in accordance with the manufacturers guidelines. The cyclists were allowed to use either a road or TT bike, but they had to use the same machine and gear ratios for each trial. Participants were instructed to adopt a position on the handlebars which was most comfortable. Using this method, 4-km laboratory TTs have previously been shown to have a coefficient of variation of 1.6 % (Altareki et al. 2009) for performance times and produce reliable pacing strategies (Stone et al. 2011) in similar participant populations. Body composition data were measured using air-displacement plethysmography (BodPod V4.2.0, Life Measurement Inc., USA). Prior to each TT, participants were instructed to abstain from any food intake in the preceding 3 h and to adhere to their normal nutritional intake strategy. Nutritional intake was recorded for the 24-h period prior to attending the initial laboratory visit, and replicated on each subsequent occasion. For the preceding 24-h period, participants were also instructed to avoid performing any exercise. Pre-exercise urine samples were obtained to measure urine osmolality using a portable osmometer (Osmocheck, Vitech Scientific, West Sussex, UK) which has previously been shown to be both valid and reliable (Sparks and Close 2013). This was to ensure that this index of hydration status was consistent prior to each trial.

### Experimental procedures

Participants performed a standardised warm-up which required them to cycle at a self-selected intensity for 5 min. This was replicated prior to each subsequent TT. Each laboratory session was performed at the same time of day to minimise the potential effects of circadian variations (Souissi et al. 2010). The first laboratory visit was a familiarisation TT as this has been shown to improve subsequent test–retest pacing strategy reliability (Ansley et al. 2004). This initial trial was followed by two experimental trials that took place between five and 7 days apart, which was chosen to limit the confounding effects of fatigue and potential changes in training status. All trials commenced 2 h after the consumption of either a 500 ml drink containing only water (Buxton still mineral water, UK), in the case of the familiarisation and the placebo trials, or after the consumption of 500 ml water containing 1000 mg of TAU (Acrōs Organics, Geel, Belgium). This pre-exercise consumption period was used to elicit peak plasma TAU concentrations at the start of the time trial (Galloway et al. 2008). The placebo and TAU TTs were conducted using a double-blind randomised crossover design. This dose was chosen because it has previously been shown to improve high intensity running performance in well-trained runners (Balshaw et al. 2013) and represents the typical dose in many commercially available energy and stimulant drinks. Data were downloaded from the ergometer software at a frequency of 37 Hz to determine each 500 m split time, speed and overall performance time.

Capillary blood samples were obtained from a finger-tip prior to the start and immediately on completion of each time trial to measure blood lactate (Lactate Pro Analyser, Arkray Inc, Kyoto, Japan), and 100 μl was collected using glass clinitubes (Radiometer Medical, Brønshøj, Denmark) to determine pO_2_, pCO_2_, pH and HCO_3_^−^ (Radiometer ABL800, Copenhagen, Denmark). Respiratory gases (Oxycon Pro, Jaeger, CareFusion, Hoechberg, Germany) and heart rate (Polar Wearlink, Polar Electro OY, Kempele, Finland) were also measured throughout each TT. Breath-by-breath respiratory responses were measured continuously during the trials which prevented participants from consuming any additional fluid during the TTs. Gas analysis data were saved using 5 s averaging at each 500 m of the trials. During the TTs, participants were only able to see the total distance covered, and were blinded to all other performance feedback and physiological variables.

### Statistical analysis

Prior to analysis, all statistical assumptions were assessed using standard graphical methods (Grafen and Hails 2002). Overall 4-km TT performance was analysed using paired *t* tests and two-way repeated measures ANOVA was used to analyse split speeds, oxygen uptake, and heart rate (condition × each 500 m of the trials). All blood sample parameters were analysed using two-way ANOVA (condition × sample point). Post hoc pair-wise comparisons were made using a Bonferroni adjustment and statistical significance was assumed as *p* < 0.05. Calculations of effect sizes were done using Cohen’s d (Cohen 1988) for t tests and partial eta squared (ηp^2^) for ANOVA. Effect sizes were considered trivial (<0.20), small (0.20–0.49), moderate (0.50–0.79), and large ≥0.80) in accordance with conventional interpretations (Cohen 1988). All data are presented as mean ± SD and with 95 % confidence intervals. All data were analysed using SPSS v22 for Windows (SPSS Inc., Chicago, IL, USA).

## Results

**Fig. 1:**
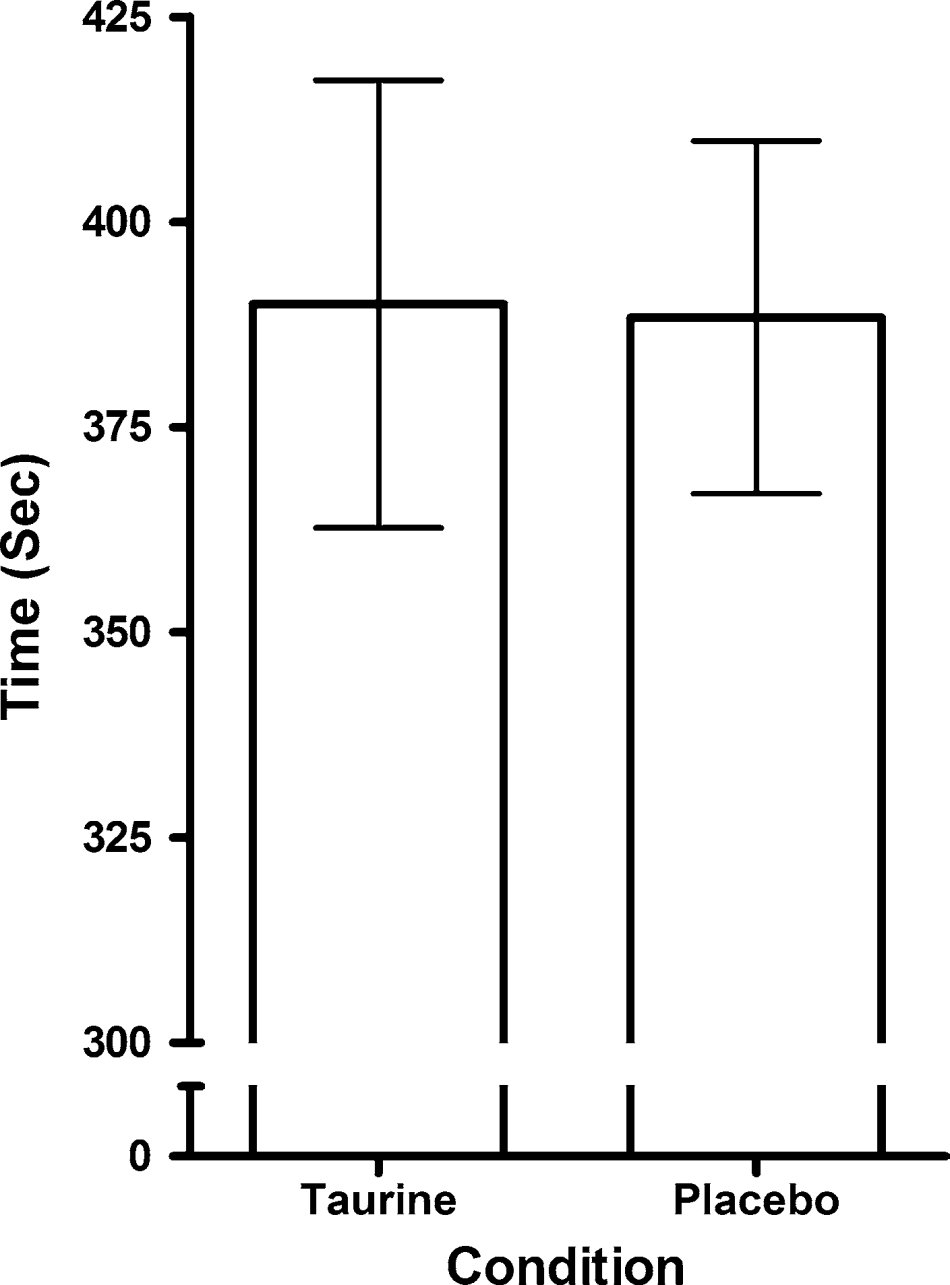
Mean (±SD) overall 4-km time trial performance times

**Fig. 2:**
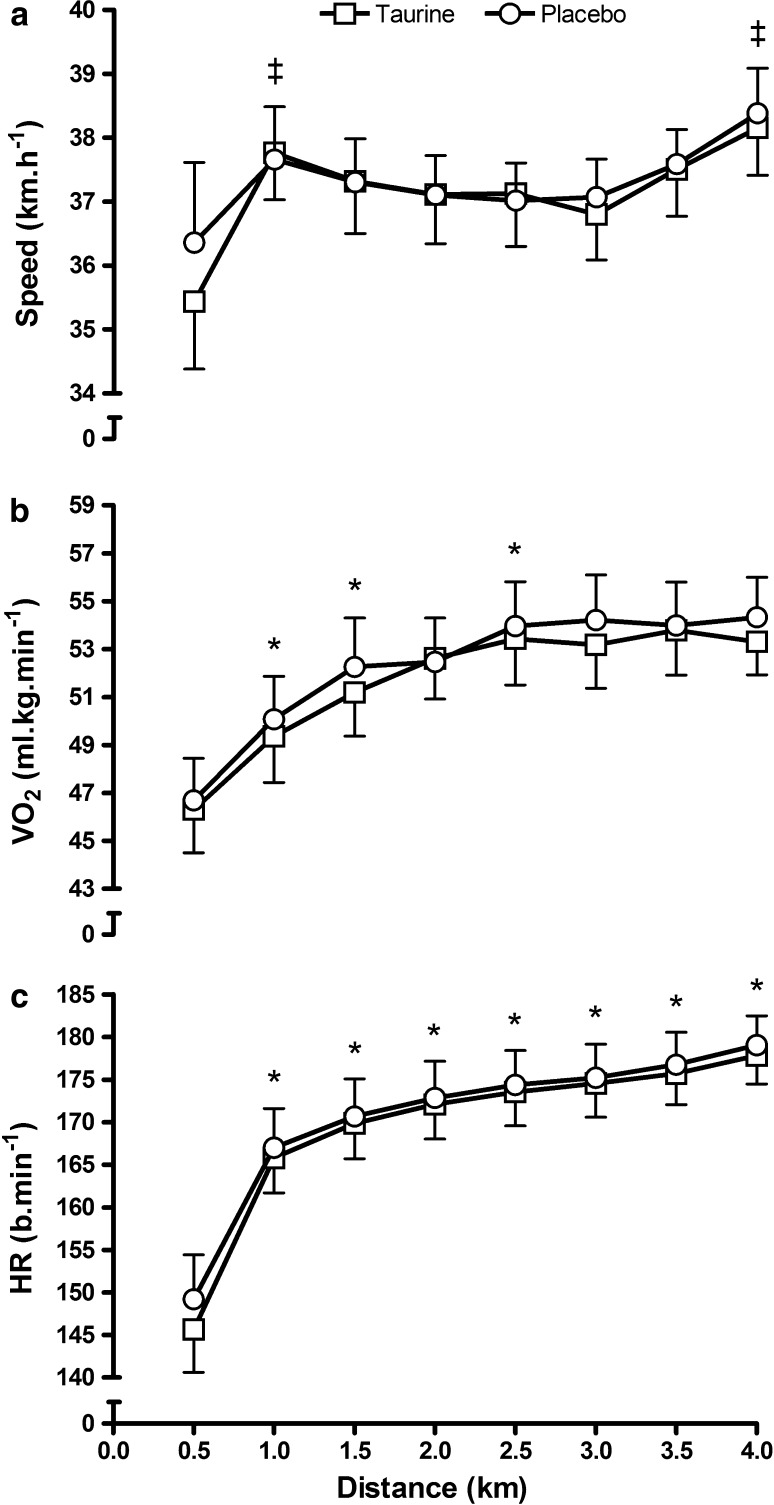
Mean (±SD) cycling speed (**a**), oxygen cost (**b**) and heart rate (**c**) responses during the time trial. ^ǂ^a significant increase in speed from previous distance (*p* < 0.05); *a significant increase from the previous distance (*p* < 0.05)

There were no statistical differences in overall performance time (Fig. 1) either between the familiarisation trial and the P trial (mean difference = 4.99 s, *t* = 0.98, CI = −6.38, 16.35, *p* = 0.35, *d* = 0.44) nor as a result of TAU ingestion (mean difference = 1.87 s, *t* = 0.35, CI = −9.89, 13.63, *p* = 0.73, *d* = 0.16). The pacing strategies adopted in the experimental trials (Fig. 2a) were characterised by increases in speed over the first kilometre (CI = −3.40, −0.23, *p* = 0.03) followed by a constant speed until 3 km, and then an acceleration during the final kilometre between (CI = −1.18, −0.27, *p* = 0.01). Despite these differences in speed over the course of the TT distance there were no main effects for experimental condition and split speeds (mean difference = 0.16 km h^−1^, *f* = 0.10, CI = −0.98, 1.30, *p* = 0.76) suggesting similar pacing strategies in both trials. The oxygen consumption (Fig. 2b) and heart rate (Fig. 2c) were also unaffected by TAU administration (mean difference = 0.60 ml kg min^−1^, *f* = 2.08, CI = −0.33, 1.53, *p* = 0.18 ηp^2^ = 0.17 and mean difference = 1.3 b min^−1^, *f* = 2.48, CI = −0.53, 3.06, *p* = 0.15, ηp^2^ = 0.20, respectively), but main effects for distance were observed for both VO_2_ (mean difference from 0.5 km to 4.0 km = 7.32 ml kg min^−1^, *f* = 26.96, CI = 5.55, 9.58, *p* < 0.001, ηp^2^ = 0.90) and heart rate (mean difference from 0.5 km to 4.0 km = 31.0 b min^−1^, *f* = 107.02, CI = 25.27, 36.72, *p* < 0.001, ηp^2^ = 0.96).

There were no main effects for experimental condition on blood lactate (mean difference = 0.19 mmol l^−1^, *f* = 1.06, CI = −0.22, 0.60, *p* = 0.33, ηp^2^ = 0.10), pO_2_ (mean difference = 0.10 kPa, *f* = 0.25, CI = −0.35, 0.56, *p* = 0.63, pη^2^ = 0.03), pCO_2_ (mean difference = 0.01 kPa, *f* = 0.01, CI = −0.26, 0.24, *p* = 0.93, ηp^2^ = 0.01), pH (mean difference = 0.003 pH units, *f* = 0.12, CI = −0.02, 0.01, *p* = 0.73, ηp^2^ = 0.010), nor HCO_3_ (mean difference = 0.17 mmol l^−1^, *f* = 0.50, CI = −0.37, 0.72, *p* = 0.50, ηp^2^ = 0.05), but there were significant main effects for time on all of the blood parameters (Table 1). This was characterised by post exercise increases in blood lactate (mean difference = 7.41 mmol l^−1^, *f* = 112.72, CI = 5.85, 8.96, *p* < 0.001, ηp^2^ = 0.92) and pO_2_ (mean difference = 2.67 kPa, *f* = 148.39, CI = 2.17, 3.17, *p* < 0.001, ηp^2^ = 0.94). Conversely pCO_2_ (mean difference = −1.04 kPa, *f* = 155.88, CI = −1.23, −0.85, *p* < 0.001, ηp^2^ = 0.94) pH (mean difference = −0.26 pH units, *f* = 77.52, CI = −0.32, −0.19, *p* < 0.001, ηp^2^ = 0.89) and HCO_3_ (mean difference = −13.47 mmol l^−1^, *f* = 334.08, CI = −15.12, −11.83, *p* < 0.001, ηp^2^ = 0.97) were all significantly lowered (Table 1) following the completion of the TTs. Base excess (Table 1) was also consequently unaffected by taurine ingestion (mean difference = 0.18 mEq l^−1^, *f* = 0.51, CI = −0.74, 0.38, *p* = 0.49, ηp^2^ = 0.05), but significant decreases were observed following the 4-km TTs (mean difference = −16.04 mEq l^−1^, *f* = 222.51, CI = −18.44, −13.65, *p* < 0.001, ηp^2^ = 0.96).

**Table 1:** Mean (±SD) pre and post time trial blood parameter responses to the experimental conditions

Parameter	Taurine	Placebo
Lactate (mmol l^−1^)		
Pre	0.9 ± 0.3	0.9 ± 0.5
Post	8.2 ± 2.4*	8.6 ± 2.0*
pO_2_ (kPa)		
Pre	9.6 ± 0.7	9.8 ± 1.1
Post	12.3 ± 0.8*	12.4 ± 0.6*
pCO_2_ (kPa)		
Pre	5.4 ± 0.3	5.3 ± 0.4
Post	4.3 ± 0.3*	4.3 ± 0.4*
pH		
Pre	7.42 ± 0.02	7.42 ± 0.01
Post	7.16 ± 0.1*	7.16 ± 0.08*
HCO_3_ (mmol l^−1^)		
Pre	25.5 ± 0.1	25.2 ± 1.6
Post	11.9 ± 2.7*	11.9 ± 2.3*
Base Excess (mEq l^−1^)		
Pre	1.29 ± 1.1	1.01 ± 1.6
Post	−14.84 ± 3.9*	−14.96 ± 3.2*

* Denotes significant post time trial changes (*p* < 0.001)

## Discussion

The aim of the present study was to determine the effects of acute oral ingestion of 1000 mg of TAU, on short duration high intensity self-paced cycling TT performance. A further aim was to assess the acid–base responses to this supplementation strategy. Previous studies investigating the use of TAU as a potential ergogenic aid have produced equivocal findings in humans (Rutherford et al. 2010; Balshaw et al. 2013), despite strong suggestions of the potential of this amino acid to influence muscle functionality during exercise. Recently, TAU administration has been demonstrated to improve running TT performance in well-trained middle distance runners (Balshaw et al. 2013) when consumed at a time that coincides with post ingestion peak plasma concentrations (Galloway et al. 2008). Balshaw et al. (2013) were, however, unable to offer direct measurements of the possible mechanisms responsible for the improvements in performance, but suggested that one role for increased plasma TAU concentrations may be as a buffer, which has also previously been demonstrated in an animal model (Nakada et al. 1991). In the present study, a 4-km cycling TT was used as a performance criterion as this produces consistent pacing strategies (Stone et al. 2011) and is reliable in trained cyclists (Ansley et al. 2004). This sensitive exercise model was used to determine any effects of 1000 mg TAU on both performance and the blood buffering capacity. Prior to and following the TTs we measured a comprehensive range of blood parameters using previously validated techniques (Cembrowski et al. 2010). As a result of these carefully selected methods, this is the first study to show that acute low dose TAU ingestion does not influence blood buffering capacity prior to, or in response to exercise. The present study was, however, unable to provide an assessment of the blood metabolite responses during exercise or in the muscle, which may provide a more complete evaluation of the dynamic responses to this type of high intensity exercise (Bangsbo et al. 1993). These exercise performance observations are in accordance with those of Rutherford et al. (2010), who used participants of a similar training status to the present study, but not those of Balshaw et al. (2013), who used athletes of a slightly higher training status, albeit for different exercise tasks. Furthermore, based on both the suggestions of Galloway et al. (2008) and the associated high concentrations of TAU in type I muscle fibres (Matsuzaki et al. 2002; Hansen et al. 2006) the lack of observed performance effects in the present study may be explained by a combination of the potentially higher proportions of these fibres in the athletes used by Balshaw et al. (2013), and the differences in training status between the participants in these studies.

The findings of the present study suggest that any observed buffering effects of TAU do not manifest in the blood in this trained cyclist population, when 1000 mg TAU is acutely ingested prior to exercise. The lack of measurable differences in buffering responses may be due to the low dose of TAU. The present findings suggest that the improved running performances observed by Balshaw et al. (2013) are unlikely to be the result of the enhanced buffering capacity which they proposed. The reasons for the discrepancy in performance between Balshaw et al. (2013) and the present study remain unclear. One suggestion might be that this TAU ingestion strategy is more effective in exercise that elicits higher lactate concentrations such as those observed by Balshaw et al. (2013), compared to the present study. Exploration of the differences between participant populations may provide some reasons for the equivocal findings in human TAU studies. Interestingly, Balshaw et al. (2013) used younger individuals (19.9 ± 1.2 vs. 34.6 ± 11.5 years) of a superior training status (all athletes ran >72 km per week and were formally coached at least twice per week), and were also more homogeneous in nature. Furthermore, in exercise studies of TAU supplementation Rutherford et al. (2010), the present study, and Galloway et al. (2008) have all used older participants (27.2 ± 1.2, 34.6 ± 11.5, and 28 ± 3 years, respectively) and found little or no effect of TAU on metabolism. One possible explanation for this may be that the lower skeletal muscle concentrations of TAU in younger individuals (Galloway et al. 2008). Yoshioka et al. (2002) observed that older rat skeletal muscle was higher in TAU content, so it is conceivable that exogenous supplementation such as that used in these exercise studies, may benefit younger individuals to a greater extent. Unfortunately, this is of course speculative, since it is at present unclear what mechanisms might be responsible for the performance increase observed by Balshaw et al. (2013).

Previously, evidence of a buffering response following intense exercise was observed in a rodent model intramuscularly, with a very high relative dose of taurine (Nakada et al. 1991). Indeed, many of the animal model studies that have observed favourable effects following taurine administration have used extremely high doses relative to body mass (Goodman et al. 2009). Shao and Hathcock (2008) have previously suggested that intakes of 3000–10,000 mg day^−1^ are safe for humans, but we chose a dose of 1000 mg as it presents an ecologically valid strategy. This dose may, however, not be sufficient enough (Hansen et al. 2006) to cause any modifications to acid–base balance (Kilding et al. 2012). The previously highest doses of pre-exercise taurine supplementation in humans has been 3000 mg acutely or 3 × 2000 mg daily (Eudy et al. 2013). Galloway et al. (2008) used an oral dose of 1.66 mg TAU and observed increased plasma taurine content of ~778 ± 139 μM, some 13 times higher than pre-ingestion concentrations 2 h later in a group of untrained participants. However, they reported that chronic dose TAU of 5000 mg day^−1^ for 7 days had no additive effect on plasma TAU concentration nor did it increase muscle TAU content. Perhaps therefore, of even more interest, may be the exploration of a method of increasing muscle TAU concentrations following ingestion, either by reducing availability, first via dietary manipulation, or by establishing a method to increase cellular uptake of exogenously supplemented TAU by the specific transporter molecule TauT (Han et al. 2006). Interestingly, with endurance training there is also an increase in p53 (Hawley and Morton 2014) which is associated with the repression of TauT (Han et al. 2006). This adds a further level of complexity to the task of understanding how training status and exercise mode might affect the effectiveness and concentration of TAU, and remains at present, unclear.

The previous work of Shao and Hathcock (2008) suggests that high doses of exogenous TAU can be safely consumed. Therefore, future work should investigate the effect of doses between 3000 and 10,000 mg day^−1^ either relative to body/lean mass, to establish if the amount of TAU ingested is a limiting factor in its effectiveness in humans. Future studies also need to establish more effective methods of manipulating muscle TAU content, or determine a specific threshold plasma concentration over which there might be an increase in uptake since the different uptake responses between rodent and human muscle might be dose dependent. Furthermore, continued work on the ingestion of this amino acid is required to determine the reasons for the largely equivocal findings in the existing data.

## Conclusion

This is the first study to show that the acute pre-exercise ingestion of 1000 mg of TAU does not affect 4-km cycling time trial performance. Furthermore, this dose did not appear to alter any of the blood buffering responses either prior to or following high intensity exercise performance in trained cyclists. This suggests that previously observed performance changes following this particular TAU ingestion strategy are unlikely to be associated with a disruption to normal acid–base balance in this population following a low dose of TAU. Future work needs to address the use of longer duration ingestion periods or exploring mechanisms by which TAU concentrations in human skeletal muscle can be increased.
